# Simultaneous FDG-PET/MRI detects hippocampal subfield metabolic differences in AD/MCI

**DOI:** 10.1038/s41598-020-69065-0

**Published:** 2020-07-21

**Authors:** Mackenzie L. Carlson, Phillip S. DiGiacomo, Audrey P. Fan, Maged Goubran, Mohammad Mehdi Khalighi, Steven Z. Chao, Minal Vasanawala, Max Wintermark, Elizabeth Mormino, Greg Zaharchuk, Michelle L. James, Michael M. Zeineh

**Affiliations:** 1 Department of Bioengineering, Stanford University, Stanford, USA; 2 Department of Radiology, Stanford University, Stanford, USA; 3 Department of Biomedical Engineering, University of California, Davis, Davis, USA; 4 Department of Neurology, University of California, Davis, Davis, USA; 5 Department of Neurology, Stanford University, Stanford, USA; 6 Nuclear Medicine Service, VA Palo Alto Health Care System, Palo Alto, USA

**Keywords:** Alzheimer's disease, Translational research, Brain imaging, Magnetic resonance imaging, Radionuclide imaging

## Abstract

The medial temporal lobe is one of the most well-studied brain regions affected by Alzheimer’s disease (AD). Although the spread of neurofibrillary pathology in the hippocampus throughout the progression of AD has been thoroughly characterized and staged using histology and other imaging techniques, it has not been precisely quantified in vivo at the subfield level using simultaneous positron emission tomography (PET) and magnetic resonance imaging (MRI). Here, we investigate alterations in metabolism and volume using [^18^F]fluoro-deoxyglucose (FDG) and simultaneous time-of-flight (TOF) PET/MRI with hippocampal subfield analysis of AD, mild cognitive impairment (MCI), and healthy subjects. We found significant structural and metabolic changes within the hippocampus that can be sensitively assessed at the subfield level in a small cohort. While no significant differences were found between groups for whole hippocampal SUVr values (p = 0.166), we found a clear delineation in SUVr between groups in the dentate gyrus (p = 0.009). Subfield analysis may be more sensitive for detecting pathological changes using PET-MRI in AD compared to global hippocampal assessment.

## Introduction

The medial temporal lobe (MTL), which includes the hippocampus and adjacent entorhinal and perirhinal cortices, is critical to memory formation and retrieval and well known to be involved in most forms of Alzheimer’s disease (AD)^1^. Neurodegeneration within the MTL thus leads to memory impairments in both AD and amnestic mild cognitive impairment (MCI)^2^. This neurodegeneration results in atrophy that can be measured using structural MRI^3^ and a reduction in metabolism of [^18^F]fluoro-deoxyglucose (FDG) in PET imaging^4^.

**Figure 1:**
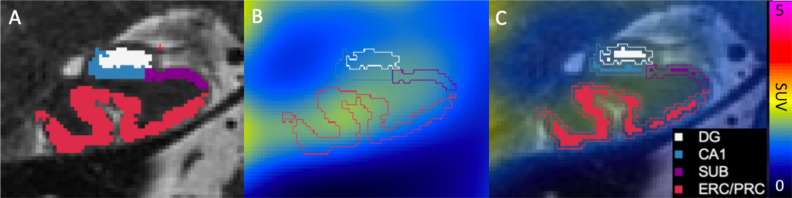
Hippocampal segmentation and erosion. In a representative AD subject, (**A**) hippocampal subfields are segmented on coronal oblique T2-w images shown at the posterior junction of the hippocampal head and body, with the subjacent posterior margin of the entorhinal cortex. (**B**) Subfields fused to SUV map. (**C**) SUV overlaid on T2-w image, with solid filled segmentation showing 1-voxel 2D erosion inside the outlined subfield area. DG encompasses the dentate gyrus and CA2-4 (because of MR resolution limitations).

The hippocampus is divided into subfields distinct in cytoarchitecture, connectivity, and function in both health and disease^5–9^. From external to internal, relevant MTL subregions include perirhinal cortex (PRC), entorhinal cortex (ERC), subiculum (SUB), cornu ammonis fields 1–4 (CA1–4), and dentate gyrus (DG) (Fig. 1). ERC connects the neocortex to the hippocampus by primarily projecting to DG, which in turn projects to CA3, then to CA1 and to SUB, and back to ERC^10^.

AD neurofibrillary pathology spreads through the hippocampus as disease progresses. In the earliest stages of AD, neurofibrillary pathology is limited to the transentorhinal (part of PRC) and entorhinal cortices^11^. As AD progresses, hippocampal involvement begins with neuron loss and tau tangles in CA1/2, progresses to CA3/4 and then to SUB and DG^12,13^. AD symptoms are strongly correlated with the extent of hippocampal neurofibrillary involvement^14,15^. Thus, image-based subfield measurements could be non-invasive biomarkers for diagnosis and staging of AD.

Several MRI studies have shown that hippocampal subfield morphological measurements detect sensitive differences between AD, MCI, and healthy subjects. Some studies have found that CA1 and DG volume is reduced in AD subjects compared to MCI subjects^14,16^ and may be a biomarker of presymptomatic AD^17–23^. Developments in MRI acquisition and processing techniques, including using high-resolution T2-weighted MR, have enabled visualization and assessment of hippocampal subfields in greater detail, including automated segmentation^15,24,25^.

FDG-PET is often used in AD as a biomarker of hypometabolism independent of amyloid and tau binding^26^. Prior to co-registered MRI and PET, most studies were unable to resolve small regions such as hippocampal subfields. Using fused non-simultaneous FDG-PET/MR imaging, studies have demonstrated hippocampal hypometabolism in AD patients^27^. While resolution limits have predominantly restricted analysis to the whole hippocampus, Choi et al*.*^28^ demonstrated that high resolution MRI fused to separately acquired FDG-PET can discern differences in FDG metabolism in manually-delineated subfields. However, non-simultaneous PET and MRI can introduce registration errors^29^, which can be avoided using simultaneous PET-MR imaging, and manual subfield delineation is prone to user-error and is not scalable.

To address the challenge of accurate MR-based delineation of hippocampal subfields and precise registration to PET to measure their metabolism, we coupled simultaneous PET-MR with automated hippocampal subfield segmentation in a cohort of AD, MCI, and healthy controls. We did not observe hypometabolism over the whole MTL when comparing AD and MCI subjects (grouped due to the small cohort size) to age-matched healthy control subjects. However, upon subfield analysis, we identified significant hypometabolism in the DG of AD and MCI subjects.

## Methods

### Subjects

We prospectively enrolled 38 subjects from our memory clinic. All experimental protocols were approved and performed in accordance with the Institutional Review Board at Stanford University and the Health Insurance Portability and Accountability Act. The Clinical Dementia Rating (CDR) scale was determined by a neurologist specializing in dementia care and was used to classify subjects. Subjects with a CDR ≥ 1 were classified as probable AD, subjects with a CDR = 0.5 were classified as MCI, and subjects with a CDR = 0 were considered healthy controls. Written informed consent was obtained from all participants or their legally authorized representative under protocols approved by the IRB at Stanford.

Data from 6 subjects were excluded from analyses due to segmentation failure (n = 2), and unsuccessful PET acquisition (n = 4), leaving 32 subjects for analysis. Of these there were 9 amnestic AD, 6 MCI (3 amnestic, 2 of whom subsequently converted to AD, and the third died still with amnestic MCI, and 3 non-amnestic, of whom only 1 subsequently converted to non-amnestic AD), and 17 age-matched control subjects (Table 1). Out of these 32 subjects, a total of 30 were included for SUVr analyses, with one control subject excluded because of poor PET signal-to-noise ratio (SNR) and one AD subject excluded due to partial volume correction error, and 30 were included for volume analyses, with one AD subject excluded because of incomplete hippocampal imaging.

**Table 1:** Subject demographics.

	AD	MCI	Healthy control
Total	9	6	17
Age ± SD	68.0 ± 7.5	70.7 ± 9.6	67.6 ± 8.2
Males/females	8/1	6/0	15/2

### Image acquisition

Subjects underwent a 75-min FDG-PET scan on a 3T PET-MR (SIGNA, GE Healthcare, WI, USA) using time-of-flight (TOF) capability, which enables faster reconstruction algorithm convergence, enhanced SNR, and more precise uptake measurements over non-TOF systems^30,31^, following a 5 mCi intravenous injection of 18F-FDG. Subjects were immobilized carefully inside the head coil and were able to cooperate by staying still during the scan, and motion correction was not applied. We collected dynamic studies with longer scan times for potential work involving estimation of cerebral metabolic rate of glucose. During the PET acquisition, we acquired a sagittal T1-weighted inversion recovery spoiled gradient echo (TR 7.6 ms, TE 3.1 ms, FA 11, 1 × 1 × 1.2 mm resolution, 5:46 min scan time) and a coronal oblique T2-weighted fast spin echo (FSE) (TR 14111 ms, TE 102 ms, FA 111, 0.43 × 0.43 × 1.9 mm resolution, 3:24 min scan time). Static 45–75-min PET TOF-OSEM reconstruction parameters were the following: 28 subsets, 3 iterations, 192 × 192 matrix, SharpIR on, standard Z-axis filter with cutoff = 4, attenuation correction, scatter correction, and deadtime correction. An MRI series with LAVA-Flex (liver-accelerated volume acquisition-Flex) based on 2-point Dixon MRI, along with an atlas-based segmentation algorithm were used to classify fat, water air, and bone in the head and then construct the attenuation correction map for each subject. This method was the best available at the time the study was conducted^32^.

### PET image-processing

Standardized uptake value (SUV) maps were calculated from PET 45–75-min summed raw data. Partial volume correction was applied to SUV maps using PETSurfer’s mri_gtmpvc algorithm with scanner point-spread function full-width/half-max = 5 mm and including auto-mask and Muller-Gartner analysis^33,34^. Although the PET-MR images are inherently co-registered, there may be misregistration due to subject motion during the PET scan, so we performed an additional fine-tuning registration step. SUV maps were thus registered to the coronal T2-weighted FSE image space using the T1-weighted scan as an intermediate registration volume and an affine transformation as implemented in NiftiReg^35^. SUV ratio (SUVr) maps were produced by normalizing the SUV maps to the pons, which was segmented in FreeSurfer 6.0 utilizing the brainstem segmentation option, after 1-pixel 3D erosion. The pons was chosen as a reference region because its metabolism and volume are least affected by disease state^36^.

### Hippocampal subfield segmentation

Hippocampal subfields were segmented based on coronal oblique T2-weighted images using Automated Segmentation of Hippocampal Subfields (ASHS)^37^ and a customized atlas^38^. Briefly, multi-atlas segmentation and label fusion with machine-learning-based error correction are combined to give consistent and accurate MTL substructure segmentations. Four subfields were included in our analyses: cornu ammonis 1 (CA1), dentate gyrus and CA2–4 as one combined subfield (DG), subiculum (SUB), and entorhinal and perirhinal cortices (BA35 and BA36) (ERC/PRC). Grouping CA2–4 and DG is a common practice in this field due to the small size of these particular subfields which limits our ability to accurately separate them^17,39,40^. The left and right subfields were combined into a single segmented volume. Automated segmentations were manually checked for accuracy by two independent raters blinded to the subject disease status.

Hippocampal subfield volumes were calculated in coronal T2-weighted FSE space, and partial-volume corrected PET images were transformed to the same space to quantify mean SUVr. In calculating mean SUVr, subfields were eroded by one voxel in 2D in the oblique coronal plane using c3d^41^ to further avoid partial volume effects (Fig. 1).

Total hippocampal volume was calculated as the sum of all four subfields. Left and right combined subfield and hippocampal mean SUVr values were derived using the subfield means weighted by the respective subfield volumes.

### Statistical analysis

Linear regression analyses were completed on the left and right-combined whole hippocampus and subfields (CA1, DG, SUB, ERC/PRC). Because the number of subjects in each group was small, AD and MCI subjects were pooled together and compared with controls using regression in MATLAB (The MathWorks, Natick, MA). p-values are reported uncorrected. A Bonferroni correction was applied to account for these five comparisons (done separately for volume and metabolism), and results meeting this corrected threshold of p < 0.01 are listed in bold in Tables 2, 3.

**Table 2:** Mean, standard deviation of volume (in mm^3^), adjusted difference (CI), and p-value comparing AD/MCI patients to control subjects in the whole hippocampus and subfields.

	AD/MCI volume in mm^3^ ± SD	Control volume in mm^3^ ± SD	Adjusted difference in mm^3^ (95% CI)	Cohen’s D	p-value
Whole HC	8,013 ± 1691	9,727 ± 1,147	− 1,630.5 (− 2,448, − 633)	d = 1.186	**0.002**
DG	1,303 ± 283	1602 ± 232	− 288.3 (− 449, − 99)	d = 1.156	**0.003**
CA1	1,810 ± 386	2,324 ± 397	− 501.0 (− 714, − 223)	d = 1.313	**0.001**
SUB	1,048 ± 193	1,204 ± 112	− 147.1 (− 247, − 34)	d = 0.989	0.011
ERC/PRC	3,852 ± 993	4,597 ± 663	− 694.1 (− 1,217, − 98)	d = 0.882	0.023

Bold indicates the p-value survives Bonferroni correction.

**Table 3:** Mean and standard deviation of SUVr for AD/MCI patients and control subjects in the whole hippocampus and subfields.

	AD/MCI SUVr ± SD	Control SUVr ± SD	SUVr difference (95% CI)	Cohen’s D	p-value
Whole HC	1.425 ± 0.245	1.508 ± 0.107	− 0.083 (− 0.231, 0.042)	d = 0.439	0.166
DG	1.236 ± 0.157	1.353 ± 0.071	− 0.116 (− 0.212, − 0.033)	d = 0.960	**0.009**
CA1	1.383 ± 0.258	1.488 ± 0.060	− 0.105 (− 0.250, 0.019)	d = 0.561	0.089
SUB	1.373 ± 0.268	1.488 ± 0.097	− 0.115 (− 0.271, 0.028)	d = 0.570	0.106
ERC/PRC	1.514 ± 0.289	1.579 ± 0.188	− 0.065 (− 0.258, 0.099)	d = 0.267	0.370

Bold indicates the p-value survives Bonferroni correction.

If significant differences were found between AD/MCI and controls in any subfield, that subfield was interrogated with linear regressions comparing AD to MCI, and MCI to controls using p < 0.05. The total intracranial volume was estimated in FreeSurfer 6.0 for each subject to account for head size in regression analyses^42^. For volume statistics, volume was used as the dependent variable with disease category (AD/MCI vs. control) as the independent variable, with estimated intracranial volume and age included as independent regressors of non-interest. For SUVr statistics, SUVr was the dependent variable, disease the independent variable, and only age was included as an independent regressor of non-interest.

## Results

### Volume

**Figure 2:**
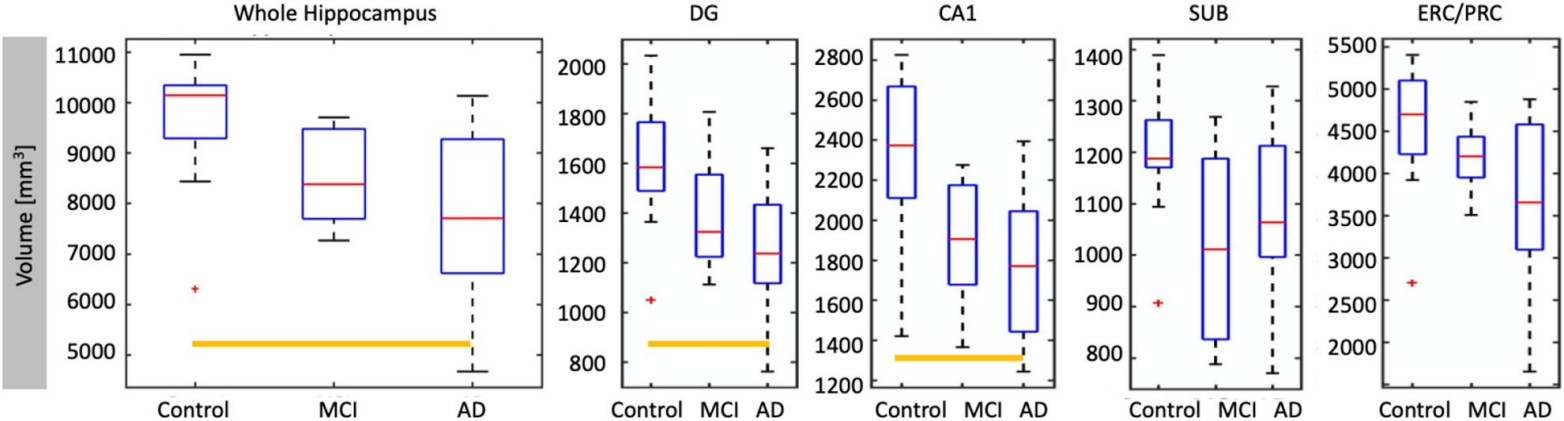
Subfield volume across cohort. Volume in combined left and right hippocampus and subfields have been plotted after partialling out estimated intracranial volume. To do this, we used residuals from a linear regression model with volume as the dependent variable and estimated intracranial volume as the independent variable, not centered about the mean. Orange bars indicate significance, and red crosses are outliers.

The combined group of AD/MCI subjects had lower left and right-combined whole hippocampal volume (p = 0.002) compared to controls. DG and CA1 subfield volumes were significantly smaller in AD/MCI compared to controls (Fig. 2, Table 2) with CA1 volume having the largest difference (p = 0.001) between groups. Volume differences between control and MCI were significant in SUB (p = 0.008) and CA1 (p = 0.029). There were no significant volume differences between AD and MCI for any subfield or whole hippocampus. Results did not change in significance in subfields or the whole hippocampus when the three non-amnestic MCI subjects were excluded from analysis.

### FDG

**Figure 3:**
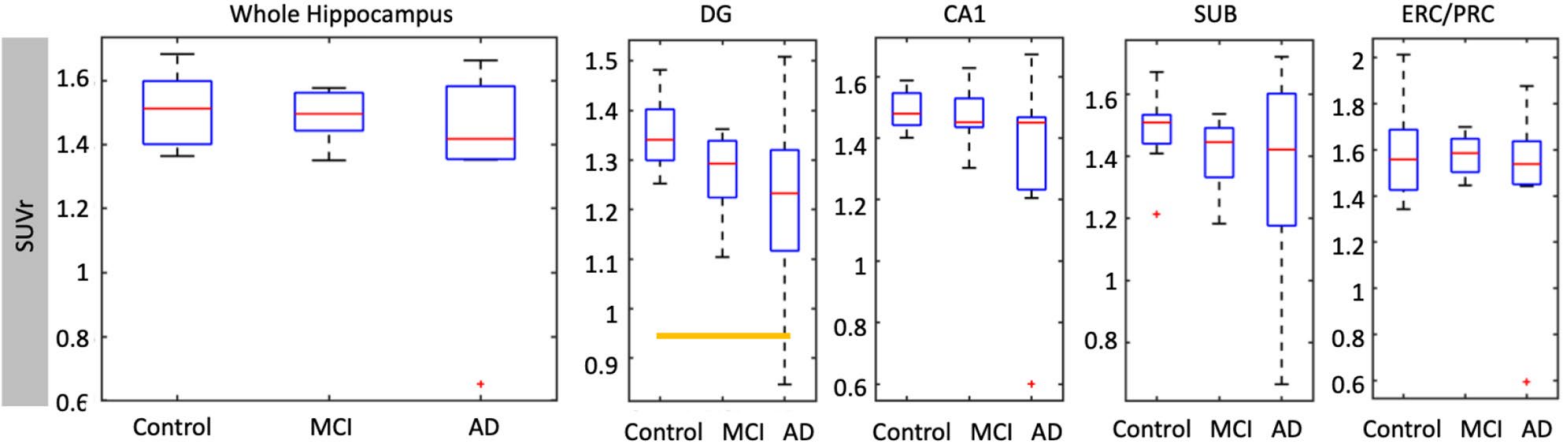
Subfield SUVr across cohort. Partial volume corrected mean SUVr (relative to pons) in combined left and right whole hippocampus and hippocampal subfields. Orange bars indicate significance, and red crosses are outliers.

Whole hippocampus SUVr was not statistically different between AD/MCI and controls (Fig. 3, Table 3, (p = 0.166). However, there was significantly lower SUVr in DG for the combined AD/MCI group compared to controls (p = 0.009). There were no significant differences in any subfield or whole hippocampus between controls and MCI, or MCI and AD. When non-amnestic MCI subjects were excluded, differences between AD/MCI and controls increased in significance in DG (p = 0.005) and trended towards but did not reach Bonferroni corrected significance in CA1 (p = 0.04), while the SUVr of other subfields and the whole hippocampus again showed no statistically significant differences.

## Discussion

By using simultaneous TOF-PET and high-resolution MRI acquisition along with automated subfield segmentation, we found significantly reduced FDG SUVr in DG and significantly reduced volume in CA1, DG, and whole hippocampus of AD/MCI patients compared to healthy controls. The SUVr difference between groups was not present when looking at the whole hippocampus and medial temporal regions. These results highlight the potential benefit of subfield-specific investigations in AD using PET/MR to observe subtle hypometabolic and atrophic effects in earlier disease stages. This approach should generalize to other tracers.

The main benefits of this imaging acquisition method are that TOF-PET/MRI enables increased SNR compared to non-TOF images, and that spatial alignment between PET and MRI images is improved using simultaneous acquisition^43^. This is especially true for long scan times, during which the subject is likely to move, and when probing small anatomical regions like hippocampal subfields and avoiding additional misregistration errors^44^. While we did not perform motion correction on this cohort, our group has since developed an optical tracking technique to correct for motion at each recorded event in list mode PET, which we will employ in future reconstructions^45^.

These advantages have allowed us to interrogate FDG metabolism in hippocampal subfields in AD, thus building upon existing FDG AD literature that has demonstrated that the whole hippocampal formation is hypometabolic in AD^27,46^, Subfield PET analysis literature is sparse, but our results suggest that hypometabolism may begin in specific hippocampal subfields before whole-hippocampal effects may be observed. We found that DG shows the highest effect of hypometabolism in AD compared to other hippocampal subfields. Park et al*.*^28^ found that AD subjects had significantly lower metabolism than healthy subjects in manually-delineated CA2/3 of both left and right hippocampi, and in CA1 and DG of the left hippocampus, which agrees with our work given that our DG delineation includes CA2 and CA3. fMRI and cerebral blood volume studies have similarly indicated that the DG shows more age-related changes than other subfields^48^. Considering that DG is one of the primary outputs of ERC—the first area affect by tau pathology^2^—via the perforant pathway, it is possible that DG hypometabolism could be driven by upstream ERC tau pathology^49^. Concordant with this idea, other AD biomarkers have shown hypometabolism, along with hippocampal atrophy, to succeed changes in CSF amyloid B levels and tau deposition in a stage-dependent manner^50,51^.

Our volumetric results are consistent with previous work, including that of Braak et al*.*^2^, which indicated that CA1 is the first hippocampal subfield atrophied in AD followed by SUB. While our measurements of DG and CA1 and whole hippocampus showed significant volume differences surviving multiple comparisons between AD/MCI and controls, only CA1 and SUB also showed significant volume differences between MCI and controls. West et al*.*^14^ also indicated that volume changes in CA1 precede entorhinal changes early on in the course of AD. Our findings corroborate other imaging studies of subfields that showed that CA1 and SUB had volume reductions with the progression of AD^15,16,23,25,52–54^.

Our results are still limited by a small sample size, so to increase statistical power we grouped AD and MCI together for our analysis. We were also unable to further stratify subject groups because we do not have a full neuropsychological assessment of memory impairment in this cohort. However, the changes in volume and metabolism are in the expected direction and magnitude given the patients’ CDR scores. Biological significance may require a larger cohort and finer stratification, but results shown here are in agreement with previous work^15,16,23,25,27,28,47,52,54,55^.

Limitations do exist in combining hippocampal subfield PET-MR. PET imaging is still limited by relatively lower spatial resolution compared to structural imaging. As a result, partial volume effects may be different among the subregions because some border white matter while others border cerebrospinal fluid and choroid plexus. Therefore, we utilized a simultaneous acquisition, further precise co-registration with MR, partial-volume correction, and subfield erosion to address this challenge. The fact that subfield metabolic differences are present in the absence of global differences may support this approach. Hippocampal subfield segmentations are currently approximate, in particular that CA2–4 are rather difficult to differentiate due to the small sizes of these regions, especially in the intricate region of the hippocampal head. In order to mitigate some of these challenges in the future, we hope to include subfield analysis at 7 T, which would allow more reliable segmentation of even smaller regions than we have shown here^56^. In addition, we would also like to correlate this subfield-level analysis with equivalent analyses using other radiotracers and larger cohorts.

## Conclusion

Taken together, we found significant structural and metabolic changes within the hippocampus that can be simultaneously assessed at the subfield level with automated techniques. Our results indicate that hypometabolic effects in the hippocampus are less pronounced than structural changes, but that subfield-specific metabolic changes begin in specific subfields before they are observable in the whole hippocampus. This study highlights the potential of PET/MRI subfield analysis for showing parallel structural and molecular disruptions accompanying dementia to obtain more nuanced imaging-based measurements for sensitive tracking and staging of disease within the hippocampus.

## References

[1] Witter MP, Amaral DG (2004). Hippocampal formation. Rat Nerv. Syst..

[2] Braak H, Braak E (1991). Neuropathological stageing of Alzheimer-related changes. Acta Neuropathol..

[3] Frisoni GB, Fox NC, Jack CR, Scheltens P, Thompson PM (2010). The clinical use of structural MRI in Alzheimer disease. Nat. Rev. Neurol..

[4] Mosconi L (2008). Hippocampal hypometabolism predicts cognitive decline from normal aging. Neurobiol. Aging.

[5] Blümcke I (2013). International consensus classification of hippocampal sclerosis in temporal lobe epilepsy: A task force report from the ILAE Commission on Diagnostic Methods. Epilepsia.

[6] Santyr BG (2017). Investigation of hippocampal substructures in focal temporal lobe epilepsy with and without hippocampal sclerosis at 7T. J. Magn. Reson. Imaging.

[7] Sapolsky RM, Uno H, Rebert CS, Finch CE (1990). Hippocampal damage associated with prolonged glucocorticoid exposure in primates. J. Neurosci..

[8] Adamowicz DH (2017). Hippocampal α-synuclein in dementia with Lewy bodies contributes to memory impairment and is consistent with spread of pathology. J. Neurosci..

[9] Goubran M (2016). In vivo MRI signatures of hippocampal subfield pathology in intractable epilepsy. Hum. Brain Mapp..

[10] Zeineh MM (2016). Direct visualization and mapping of the spatial course of fiber tracts at microscopic resolution in the human hippocampus. Cereb. Cortex.

[11] Braak H, Alafuzoff I, Arzberger T, Kretzschmar H, Del Tredici K (2006). Staging of Alzheimer disease-associated neurofibrillary pathology using paraffin sections and immunocytochemistry. Acta Neuropathol..

[12] Rössler M, Zarski R, Bohl J, Ohm TG (2002). Stage-dependent and sector-specific neuronal loss in hippocampus during Alzheimer’s disease. Acta Neuropathol..

[13] Schönheit B, Zarski R, Ohm TG (2004). Spatial and temporal relationships between plaques and tangles in Alzheimer-pathology. Neurobiol. Aging.

[14] West MJ, Coleman PD, Flood DG, Troncoso JC (1994). Differences in the pattern of hippocampal neuronal loss in normal ageing and Alzheimer’s disease. Lancet.

[15] Mueller SG (2010). Hippocampal atrophy patterns in mild cognitive impairment and Alzheimer’s disease. Hum. Brain Mapp..

[16] La Joie R (2013). Hippocampal subfield volumetry in mild cognitive impairment, Alzheimer’s disease and semantic dementia. NeuroImage Clin..

[17] Csernansky JG (2005). Preclinical detection of Alzheimer’s disease: Hippocampal shape and volume predict dementia onset in the elderly. Neuroimage.

[18] Scher AI (2007). Hippocampal shape analysis in Alzheimer’s disease: A population-based study. Neuroimage.

[19] Frisoni GB (2008). Mapping local hippocampal changes in Alzheimer’s disease and normal ageing with MRI at 3 Tesla. Brain.

[20] Gerardin E (2009). Multidimensional classification of hippocampal shape features discriminates Alzheimer’s disease and mild cognitive impairment from normal aging. Neuroimage.

[21] Raji CA, Lopez OL, Kuller LH, Carmichael OT, Becker JT (2009). Age, Alzheimer disease, and brain structure. Neurology.

[22] Yassa MA (2010). High-resolution structural and functional MRI of hippocampal CA3 and dentate gyrus in patients with amnestic mild cognitive impairment. Neuroimage.

[23] Apostolova LG (2006). Conversion of mild cognitive impairment to Alzheimer disease predicted by hippocampal atrophy maps. Arch. Neurol..

[24] Pluta J, Yushkevich P, Das S, Wolk D (2012). In vivo analysis of hippocampal subfield atrophy in mild cognitive impairment via semi-automatic segmentation of T2-weighted MRI. J. Alzheimer’s Dis..

[25] Wisse LEM, Biessels GJ, Geerlings MI (2014). A critical appraisal of the hippocampal subfield segmentation package in FreeSurfer. Front. Aging Neurosci..

[26] Ou Y-N (2019). FDG-PET as an independent biomarker for Alzheimer’s biological diagnosis: A longitudinal study. Alzheimers Res. Ther..

[27] Mosconi L (2005). Reduced hippocampal metabolism in MCI and AD. Neurology.

[28] Choi E-J (2018). Glucose hypometabolism in hippocampal subdivisions in Alzheimer’s disease: A pilot study using high-resolution 18F-FDG PET and 7.0-T MRI. J. Clin. Neurol..

[29] Kiebel SJ, Ashburner J, Poline J-B, Friston KJ (1997). MRI and PET coregistration: A cross validation of statistical parametric mapping and automated image registration. Neuroimage.

[30] Surti S (2015). Update on time-of-flight PET imaging. J. Nucl. Med..

[31] Vandenberghe S, Mikhaylova E, D’Hoe E, Mollet P, Karp JS (2016). Recent developments in time-of-flight PET. EJNMMI Phys..

[32] Sekine T (2016). Evaluation of atlas-based attenuation correction for integrated PET/MR in human brain: Application of a head atlas and comparison to true CT-based attenuation correction. J. Nucl. Med..

[33] Greve DN (2014). Cortical surface-based analysis reduces bias and variance in kinetic modeling of brain PET data. Neuroimage.

[34] Greve DN (2016). Different partial volume correction methods lead to different conclusions: An 18F-FDG-PET study of aging. Neuroimage.

[35] Modat M (2010). Fast free-form deformation using graphics processing units. Comput. Methods Progr. Biomed..

[36] Minoshima S, Frey KA, Foster NL, Kuhl DE (1995). Preserved pontine glucose metabolism in Alzheimer disease: a reference region for functional brain image (PET) analysis. J. Comput. Assist. Tomogr..

[37] Yushkevich PA (2010). Nearly automatic segmentation of hippocampal subfields in in vivo focal T2-weighted MRI. Neuroimage.

[38] Parivash SN (2019). Longitudinal changes in hippocampal subfield volume associated with collegiate football. J. Neurotrauma.

[39] Wang L (2006). Abnormalities of hippocampal surface structure in very mild dementia of the Alzheimer type. Neuroimage.

[40] Christensen A (2015). Hippocampal subfield surface deformity in nonsemantic primary progressive aphasia. Alzheimer’s Dement. Diagn. Assess. Dis. Monit..

[41] Yushkevich PA (2006). User-guided 3D active contour segmentation of anatomical structures: Significantly improved efficiency and reliability. Neuroimage.

[42] Fischl B (2012). FreeSurfer. Neuroimage.

[43] Monti S (2017). An evaluation of the benefits of simultaneous acquisition on PET/MR coregistration in head/neck imaging. J. Healthc. Eng..

[44] Metere R, Kober T, Möller HE, Schäfer A (2017). Simultaneous quantitative MRI mapping of T1, T2* and magnetic susceptibility with multi-echo MP2RAGE. PLoS ONE.

[45] Spangler-Bickell MG (2019). Rigid motion correction for brain PET/MR imaging using optical tracking. IEEE Trans. Radiat. Plasma Med. Sci..

[46] De Santi S (2001). Hippocampal formation glucose metabolism and volume losses in MCI and AD. Neurobiol. Aging.

[47] Small SA, Chawla MK, Buonocore M, Rapp PR, Barnes CA (2004). Imaging correlates of brain function in monkeys and rats isolates a hippocampal subregion differentially vulnerable to aging. Proc. Natl. Acad. Sci. U. S. A..

[48] Tiddens HAWM, Stick SM, Davis S (2014). Multi-modality monitoring of cystic fibrosis lung disease: The role of chest computed tomography. Paediatr. Respir. Rev..

[49] Hyman BT, Van Hoesen GW, Damasio AR, Barnes CL (1984). Alzheimer’s disease: Cell-specific pathology isolates the hippocampal formation. Science (80-)..

[50] Lo RY (2011). Longitudinal change of biomarkers in cognitive decline. Arch. Neurol..

[51] Jack CR (2013). Tracking pathophysiological processes in Alzheimer’s disease: An updated hypothetical model of dynamic biomarkers. Lancet. Neurol..

[52] Zhao W (2019). Trajectories of the hippocampal subfields atrophy in the Alzheimer’s disease: A structural imaging study. Front. Neuroinform..

[53] Carlesimo GA (2015). Atrophy of presubiculum and subiculum is the earliest hippocampal anatomical marker of Alzheimer’s disease. Alzheimer’s Dement. Diagn. Assess. Dis. Monit..

[54] Scelsi MA, Iglesias E, Schott JM, Ourselin S, Altmann A (2017). The role of hippocampal subfields in the atrophy process in Alzheimer’s disease: An in-vivo study of the ADNI cohort. Alzheimer’s Dement..

[55] Small SA (2014). Isolating pathogenic mechanisms embedded within the hippocampal circuit through regional vulnerability. Neuron.

[56] Parekh MB, Rutt BK, Purcell R, Chen Y, Zeineh MM (2015). Ultra-high resolution in-vivo 7.0T structural imaging of the human hippocampus reveals the endfolial pathway. Neuroimage.

